# IL‐37 Mitigates the Inflammatory Response in Macrophages Induced by SARS‐CoV‐2 Omicron Infection Through the NF‐κB Signaling Pathway

**DOI:** 10.1002/mco2.70229

**Published:** 2025-05-31

**Authors:** Feifei Qi, Yiwei Yan, Mingya Liu, Qi Lv, Yanfeng Xu, Ming Liu, Fengdi Li, Ran Deng, Xujian Liang, Shuyue Li, Guocui Mou, Linlin Bao

**Affiliations:** ^1^ Beijing Key Laboratory for Animal Models of Emerging and Reemerging Infectious Diseases, NHC Key Laboratory of Comparative Medicine, Institute of Laboratory Animal Science, CAMS &PUMC Beijing China; ^2^ National Center of Technology Innovation for Animal Model Beijing China; ^3^ State Key Laboratory of Respiratory Health and Multimorbidity Beijing China

**Keywords:** IL‐37, inflammatory response, macrophages, NF‐κB, omicron

## Abstract

The expression levels of macrophage‐associated cytokines are significantly greater in COVID‐19 patients than in healthy individuals. Exploring strategies to modulate pathological cytokine storms can effectively prevent the development of severe coronavirus infection‐induced pneumonia. Treatment with interleukin‐37 (IL‐37), an anti‐inflammatory factor, has unique anti‐inflammatory and antiviral effects on infections caused by various pathogens. In this study, we investigated the effect of IL‐37 treatment on the SARS‐CoV‐2 Omicron‐infection induced inflammatory response and its molecular mechanism. Our results demonstrated that IL‐37 treatment effectively alleviated symptoms, reduced viral loads, suppressed the production of proinflammatory cytokines and chemokines both systemically (in serum) and locally (in the lungs), and attenuated lung lesions and inflammatory cell infiltration in Omicron‐infected mice. The suppressed proinflammatory factors were macrophage‐related, particularly CCL3 and CCL4, which were significantly inhibited. Furthermore, treatment with IL‐37 significantly reduced the proportion of M1‐type macrophages in lungs of Omicron‐infected mice. In addition, we found that IL‐37 targeted M1 macrophages through modulation of the NF‐κB signaling pathway to suppress the production of proinflammtory factors during Omicron infection. This study elucidated the anti‐inflammatory effect of IL‐37 treatment on the Omicron‐induced inflammatory response while identifying its specific target site, thereby providing fundamental insights for exploring potential clinical therapeutic interventions.

## Graphical abstract

IL‐37 mitigates the infiltration of macrophages and suppresses the production of macrophage‐associated chemokines (such as CCL3 and CCL4) induced by SARS‐CoV‐2 Omicron infection through NF‐κB signaling pathway.

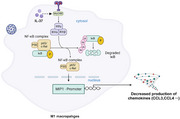

## 1 Introduction

High viral loads of severe acute respiratory syndrome coronavirus 2 (SARS‐CoV‐2) can enhance type I interferon (IFN‐I) responses, induce various cytokines production, and activate neutrophils, monocytes, macrophages, and other inflammatory cells, thereby playing an immunomodulatory role [1, 2]. Compared with mild patients, patients with severe COVID‐19 exhibit pronounced infiltration of macrophages in the lungs and significantly elevated production of macrophage‐associated inflammatory factors, including plasma C‐C motif chemokine ligand 3 (CCL3), C‐X‐C motif chemokine 10 (CXCL10), and CCL2 [3, 4]. Studies have demonstrated comparable levels of SARS‐CoV‐2 viral load in throat swabs among patients with asymptomatic, mild, and severe COVID‐19; however, elevated plasma concentrations of inflammatory factors such as interleukin‐1 beta (IL‐1β) and interferon‐gamma (IFN‐γ) are observed in patients with severe disease compared to those with mild disease [5]. Similarly, SARS‐CoV infection leads to the upregulation of chemokines such as CXCL10 and CCL2, leading to the recruitment of inflammatory cells and exacerbating the severity of SARS [6, 7].

Likewise, a significant presence of infiltrating macrophages is observed in the lungs of middle east respiratory syndrome (MERS) patients who succumb to this disease, accompanied by elevated levels of proinflammatory factors, including serum interleukin‐6 (IL‐6), interferon‐alpha (IFN‐α), and CXCL10 [8]. In a similar fashion, patients with severe H7N9 exhibit a substantial number of activated macrophages in their lungs and elevated expression of inflammatory factors such as serum IL‐6, interleukin‐8 (IL‐8), and CCL4 [9], suggesting that respiratory infection‐induced production of inflammatory factors associated with the activation of inflammatory cells, such as macrophages, is associated with the severity of disease.

Therapeutic regimens that effectively balance the immune response while mitigating pathological cytokine storms are pivotal for the clinical prevention and treatment of coronavirus infection. The administration of antiviral drugs during the early stages of SARS‐CoV‐2 infection can impede viral replication and minimize tissue damage; however, it is insufficient to counteract cytokine storms in the later stages of the disease. Consequently, additional targeting of the inflammatory response is imperative to improve the disease prognosis [10, 11]. Early administration of aspirin, a nonsteroidal anti‐inflammatory drug, has been shown to reduce in‐hospital mortality in severe COVID‐19 patients [12]; however, high‐dose aspirin may inhibit the synthesis of cyclooxygenase‐2 and prostacyclin in endothelial cells, exacerbating endothelial dysfunction and increasing the risk of hemorrhage [13]. Additionally, the administration of tocilizumab, an inhibitor of the IL‐6 receptor signaling pathway, improves discharge rates among critically ill COVID‐19 patients but fails to reduce all‐cause mortality and increases the risk of secondary infections [14]. Although antiviral drugs alleviate early clinical symptoms in COVID‐19 patients, their impact on prognosis remains limited for newly diagnosed individuals. Furthermore, most anti‐inflammatory drugs targeting patients with severe COVID‐19 have potentially serious side effects. Therefore, further investigations are warranted to identify therapeutic strategies for COVID‐19 that effectively balance the risk of cytokine storms and side effects.

IL‐37 is distinguished by its unique role as a natural inhibitor of inflammatory responses, effectively suppressing T‐cell activation, dendritic cell maturation, and the production of proinflammatory cytokines [15, 16]. Through these mechanisms, IL‐37 has emerged as a potent regulator that can restore balance to an overactive immune system. Similarly, IL‐37 exhibits distinctive anti‐inflammatory effects against various pathogens, including influenza virus, MERS‐CoV, coxsackievirus B3, and *Mycobacterium tuberculosis* [17, 18, 19, 20, 21]. IL‐37 effectively suppresses viral replication in H3N2‐infected A549 cells [17]. IL‐37 treatment enhances survival and reduces lung inflammation in H1N1‐infected mice, accompanied by a significant reduction in CCL2, IL‐1β, IL‐6, and CXCL10 in the lungs [18]. Coxsackievirus B3 infection induces myocardial injury and impaired cardiac function in mice, and treatment with IL‐37 increases the survival rate of infected mice, attenuates cardiac inflammatory cell infiltration and collagen fiber deposition, and suppresses the expression of inflammatory factors such as serum IL‐1β and interleukin‐18 (IL‐18) [19]. Additionally, IL‐37 treatment attenuates hepatic injury and inhibits the expression of IL‐1β, IL‐6, interleukin‐10 (IL‐10), and interleukin‐4 (IL‐4) in the liver tissues of cytomegalovirus‐infected mice [20]. Similarly, the expression of inflammatory factors such as IL‐6, tumor necrosis factor‐α (TNF‐α), and IL‐1β is reduced by 70% in RAW264.7 cells transfected with human IL‐37. Furthermore, bone marrow‐derived macrophages (BMDMs) from IL‐37 transgenic mice exhibit a significant reduction in the expression of inflammatory factors, including IL‐6, IL‐1β, IL‐1α, and TNF‐α [22]. These studies collectively demonstrate the inhibitory effects of IL‐37 on inflammatory cytokine production, attenuation of the inflammatory response, and its anti‐inflammatory effects on diseases resulting from infections caused by various pathogens. Furthermore, investigations have shown that compared with healthy individuals, COVID‐19 patients exhibit elevated serum IL‐37 levels. Notably, COVID‐19 patients with heightened serum IL‐37 concentrations have decreased levels of inflammatory cytokines such as IL‐6 and IL‐8, along with improved clinical outcomes, including a shorter hospitalization duration, faster viral nucleic acid clearance time, and expedited resolution of coughing [23]. Consequently, IL‐37 may suppress inflammation, inhibit disease progression, and protect against SARS‐CoV‐2 infection.

In this study, we utilized a mouse model of SARS‐CoV‐2 infection to investigate the inhibitory role of IL‐37 in pneumonia caused by SARS‐CoV‐2 infection and explored the target cells and molecular mechanisms to identify novel therapeutic strategies for the clinical prevention and treatment of COVID‐19.

## 2 Results

### 2.1 IL‐37 Intervention Mitigated the Symptoms of Omicron Infection

**FIGURE 1:**
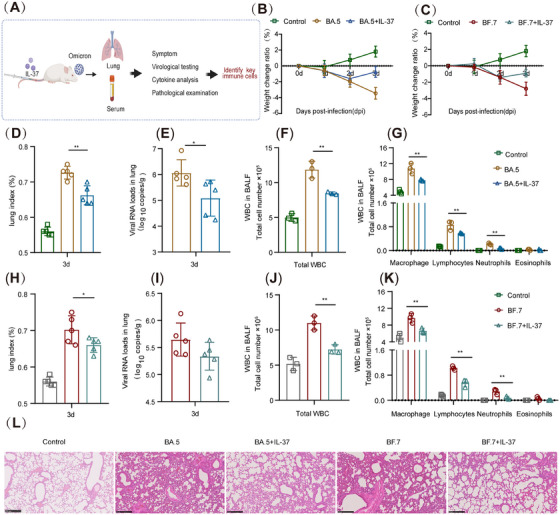
IL‐37 plays an anti‐inflammatory role in Omicron‐induced pneumonia. (A) Experimental design and sample collection. (B and C) Changes in the weights of mice infected with the Omicron BA.5 or BF.7 strain in the presence or absence of IL‐37. (D and H) Lung index of mice infected with the BA.5 or BF.7 strain in the presence or absence of IL‐37. (E and I) Viral loads in the lungs of mice infected with the BA.5 or BF.7 strain in the presence or absence of IL‐37. (F, G, J, and K) Total number of WBCs in BALF from mice infected with the BA.5 or BF.7 strain in the presence or absence of IL‐37. (L) Histopathology of lung tissues from mice infected with Omicron. *p < 0.05, **p < 0.01.

The experimental design and sample collection conditions are shown in Figure 1A. The weights of the control mice continued to increase, with a 1.8% weight gain at 3 days post infection (dpi) (Figure 1B,C). The model mice exhibited a continuous decrease in weight for 3 days after BA.5 or BF.7 infection, and then the weights of the BA.5+IL‐37 and BF.7+IL‐37 mice began to rebound at 3 dpi (Figure 1B,C). The average lung index in the BA.5‐infected mice was 0.73, which decreased to 0.66 following IL‐37 treatment (Figure 1D). In the BF.7 model group, the mean lung index was 0.70, and it decreased to 0.66 after IL‐37 treatment (Figure 1H). Additionally, there was a one‐log decrease in the average viral load in the BA.5+IL‐37 group and a 0.3‐log decrease in the BF.7+IL‐37 group (Figure 1E,I). The total number of WBCs and specific cell populations in the Bronchoalveolar lavage fluid (BALF) were identified and are presented in Figure 1F,G,J,K; the results revealed a significant decrease in the total cell count, as well as in the numbers of macrophages, lymphocytes, and neutrophils in the BA.5+IL‐37 and BF.7+IL‐37 groups compared with those in the BA.5 and BF.7 groups. The lung tissues of the mice in the BA.5 model group and the BF.7 model group presented moderate interstitial pneumonia, the alveolar interval was significantly widened, and many inflammatory cells infiltrated the blood vessels and bronchi. In the BA.5+IL‐37 group, two of five mice had moderate interstitial pneumonia, and three of five had mild interstitial pneumonia. In the BF.7+IL‐37 group, one of five mice had moderate interstitial pneumonia, and four of five had mild interstitial pneumonia. In the IL‐37 treatment groups, the lung tissue inflammation was significantly reduced, the alveolar interval was slightly widened, inflammatory cell infiltration was relieved, and perivascular inflammatory cell infiltration was reduced (Figure 1L).

### 2.2 IL‐37 Treatment Alleviated Lung Inflammation in Omicron‐Infected Mice

**FIGURE 2:**
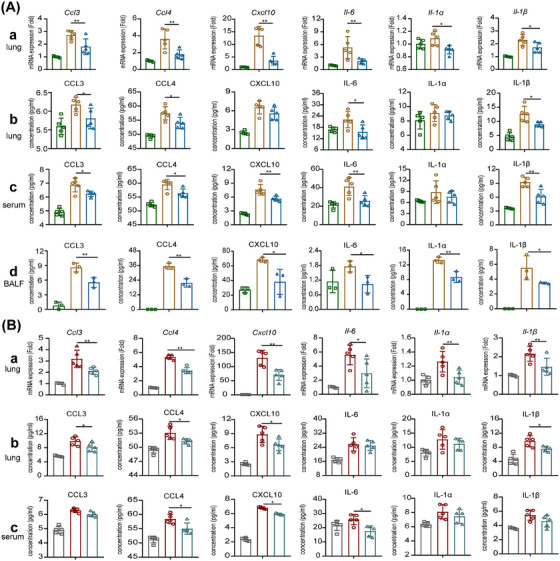
IL‐37 treatment inhibited the production of pro‐inflammatory factors in Omicron‐infected mice. (A‐a) Relative mRNA expression of cytokines and chemokines in the lung tissues of BA.5‐infected mice with/without IL‐37 treatment. (A‐b) Protein production of cytokines and chemokines in the lung tissues of BA.5‐infected mice with/without IL‐37 treatment. (A‐c) Protein production of cytokines and chemokines in the serum of BA.5‐infected mice with/without IL‐37 treatment. (A‐d) Protein production of cytokines and chemokines in BALF of BA.5‐infected mice with/without IL‐37 treatment. (B‐a) Relative mRNA expression of cytokines and chemokines in the lung tissues of BF.7‐infected mice with/without IL‐37 treatment. (B‐b) Protein production of cytokines and chemokines in the lung tissues of BA.5‐infected mice with/without IL‐37 treatment. (B‐c) Protein production of cytokines and chemokines in the serum of BA.5‐infected mice with/without IL‐37 treatment. *p < 0.05, **p < 0.01.

To further explore the anti‐inflammatory effect of IL‐37 in Omicron‐induced pneumonia, lung tissues were collected to evaluate the mRNA and protein expression levels of inflammatory cytokines. As depicted in Figure 2A‐a,B‐a, IL‐37 treatment markedly decreased the mRNA expression of macrophage‐associated cytokines and chemokines, including *Ccl3*, *Ccl4, Cxcl10*, *Il‐6, Il‐1α*, and *Il‐1β* in lung tissues. Furthermore, a significant reduction in the production of chemokine and cytokines proteins, such as CCL3, CCL4, IL‐6, and IL‐1β, were observed in the lung tissues of the BA.5+IL‐37 mice. Similarly, the production of CCL3, CCL4, CXCL10, and IL‐1β was significantly diminished in the lung tissues of the BF.7+IL‐37 group (Figure 2A‐b,B‐b). Additionally, the content of CXCL10, IL‐1α, and IL‐1β was substantially lower in the BALF from BA.5+IL‐37 mice than in that from BA.5 mice (Figure 2A‐d). Likewise, IL‐37 administration significantly decreased the serum levels of CCL3, CCL4, CXCL10, and IL‐6, in both the BA.5+IL‐37 and BF.7+IL‐37 groups (Figure 2A‐c,B‐c). These findings, when considered collectively, provide substantial evidence that IL‐37 intervention plays a crucial role in reducing the secretion of proinflammatory factors, especially the release of the chemokine CCL3 and CCL4 triggered by Omicron infection.

### 2.3 IL‐37 Treatment Alleviates Macrophage Infiltration in Lung Tissue During Omicron Infection

**FIGURE 3:**
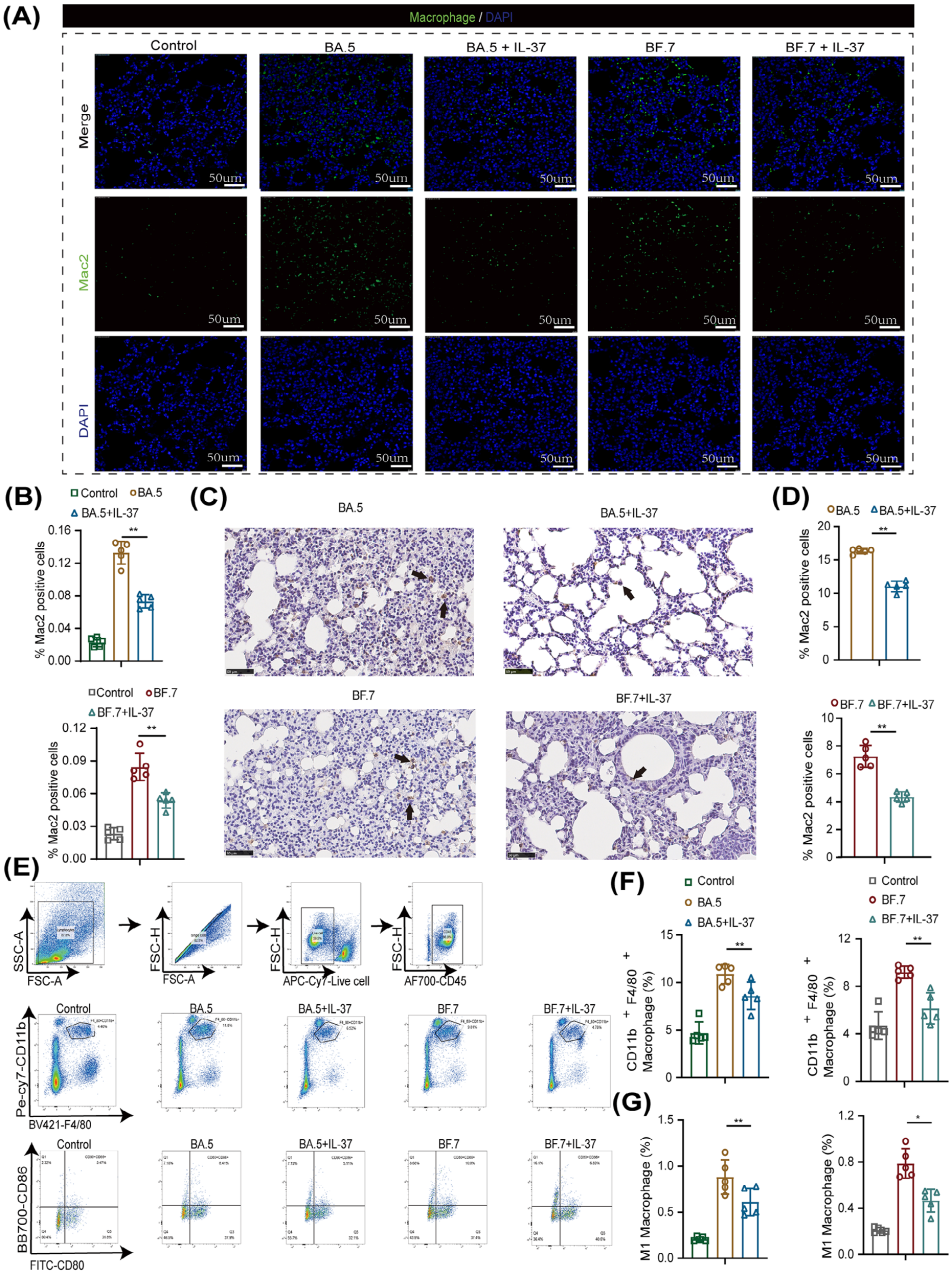
IL‐37 treatment reduces the percentage of macrophages in the lungs during Omicron infection. (A) Macrophages in mouse lung tissue were stained by immunofluorescence. The macrophages were stained with a specific anti‐Mac2 antibody (green), and the nuclei were stained with DAPI (blue). (magnification×400, white bar = 50 µm) (B) Quantitative analysis of the results shown in (A). (C) Macrophages in mouse lung tissue were stained by immunohistochemistry. The arrow marks the brown particle as the macrophage marker Mac2. (Magnification×400, black bar = 50 µm) (D) Quantitative analysis of the results shown in (C). (E) The gating strategy for identifying macrophages (CD45^+^CD11b^+^F4/80^+^) and M1 macrophages (CD45^+^CD11b^+^F4/80^+^CD80^+^CD86^+^) in mouse lung tissue via flow cytometry. (F and G) Quantitative analysis was conducted of the results of (E). *p < 0.05, **p < 0.01.

The administration of IL‐37 significantly attenuated the expression levels of cytokines and chemokines induced by Omicron infection, particularly by suppressing the production of macrophage‐associated inflammatory factors. To further investigate this phenomenon, we examined the distribution and proportion of macrophages in mouse lung tissues using immunofluorescence and immunohistochemical staining. The results revealed that the proportion of Mac‐2^+^ macrophages in the lungs significantly increased in mice infected with the Omicron BA.5 and BF.7 strains (Figure 3A–D). This indicates that Omicron infection triggers the activation of macrophages, particularly inflammatory macrophages, thereby contributing to an enhanced immune response. To further explore the macrophage subtypes affected by IL‐37, we stained M1 macrophages with antibodies against CD80 and CD86, and M2 macrophages with antibody against CD206 by flow cytometry (Figure 3E). The proportion of macrophages, particularly M1 macrophages, was significantly increased in lungs of Omicron BA.5‐ and BF.7‐infected mice (Figure 3G), further suggesting that M1 polarization macrophage plays a critical pro‐inflammatory role during Omicron infection.

In addition, there was a significant decrease in the proportion of M1 macrophages in the lungs of mice following intravenous administration of IL‐37 (Figure 3F,G). Compared with those in the model group, the proportions of M1 macrophages in both the BA.5+IL‐37‐treated group and the BF.7+IL‐37‐treated group were reduced to 70% (Figure 3F,G). However, the proportion of M2 macrophages in the lungs of Omicron BA.5‐ and BF.7‐infected mice did not change significantly before and after IL‐37 administration (data not shown). These findings suggest that IL‐37 mitigates lung tissue inflammation induced by Omicron infection through the modulation of macrophage infiltration, particularly that of M1 macrophages.

### 2.4 Inhalation of IL‐37 Effectively Mitigates the Inflammatory Response Induced by Infection With Omicron

**FIGURE 4:**
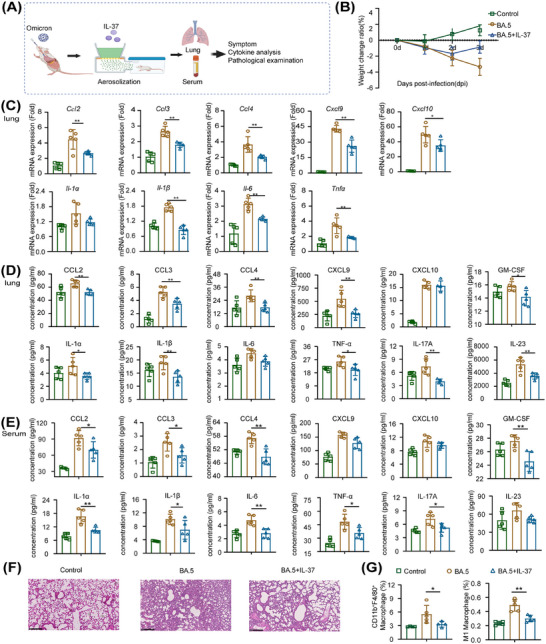
Aerosolized IL‐37 plays an anti‐inflammatory role in Omicron‐induced pneumonia. (A) Experimental design and sample collection. (B) Changes in weight change of mice infected with the BA.5 strain with or without nebulized IL‐37 administration. (C) Relative mRNA expression of cytokines and chemokines in the lung tissues of BA.5‐infected mice with or without nebulized IL‐37 administration. (D) Protein production of inflammatory factors in the lung tissues of BA.5‐infected mice with or without nebulized IL‐37 administration. (E) Protein expression of cytokines and chemokines in the serum of BA.5‐infected mice with or without IL‐37‐nebulized administration. (F) Histopathology of lung tissues from IL‐37‐treated mice infected with BA.5. (G) The proportions of macrophages and M1 macrophages in BA.5‐infected mice were assessed via flow cytometry after IL‐37 nebulized administration. *p < 0.05, **p < 0.01.

To investigate the effects of the mode of IL‐37 administration, we administered IL‐37 via nebulization to BA.5‐infected mice to test whether nebulized administration could exert comparable anti‐inflammatory effects those of intravenous administration. The experimental design and sample collection are depicted in Figure 4A. The findings demonstrated a continuous decrease in the body weights of the mice in the BA.5 model group. In contrast, mice receiving nebulized IL‐37 exhibited a 1.7% reduction in body weight at 2 dpi, followed by a rebound at 3 dpi, with a loss rate of approximately 0.9% (Figure 4B). Additionally, there was a significant decrease in the mRNA expression of proinflammatory factors, including *Ccl2*, *Ccl3*, *Ccl4*, *Cxcl9*, *Cxcl10*, *Il‐1α*, *Il‐1β*, *Tnfα*, and *Il‐6*, in the lung tissues of the mice after nebulized IL‐37 administration (Figure 4C). Notably, the production of cytokines and chemokines in the lung tissues, including CCL2, CCL3, CCL4, CXCL9, GM‐CSF, IL‐1α, IL‐1β, TNF‐α, interleukin‐17A (IL‐17A), and IL‐23, in the lungs of the IL‐37‐treated mice was notably decreased (Figure 4D). Furthermore, the production of the same proinflammatory factors in the serum was also significantly lower in the BA.5+IL‐37 group than in the BA.5‐infected group (Figure 4E). Pathology revealed moderate interstitial pneumonitis in four of five BA.5 model group mice, whereas mild interstitial pneumonitis occurred in one case. Among the nebulized IL‐37‐treated group mice, moderate interstitial pneumonitis was observed in three out of five cases, whereas mild interstitial pneumonitis occurred in two cases, indicating reduced lung inflammation and alleviation of inflammatory cell infiltration due to the inhibitory effect of nebulized IL‐37 administration on inflammation caused by BA.5 infection (Figure 4F). Moreover, the proportions of macrophages and M1 macrophages decreased significantly in the lungs of the mice in the IL‐37 administration group (Figure 4G). These results suggest that inhalation of IL‐37 can ameliorate symptoms and mitigate the inflammatory response induced by Omicron infection.

### 2.5 IL‐37 Treatment Inhibits the Expression of Inflammatory Factors in M1 Macrophages Infected With Omicron

**FIGURE 5:**
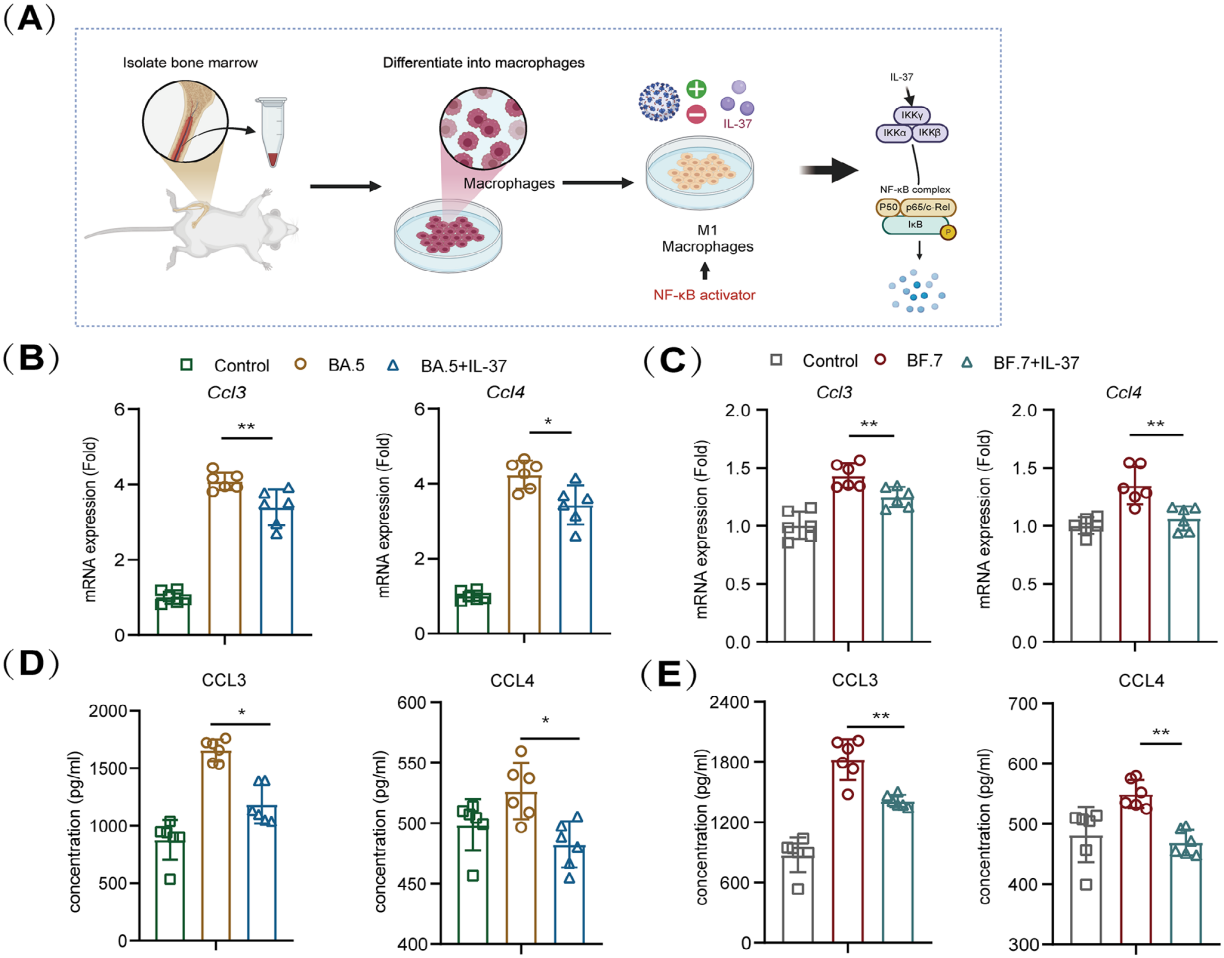
IL‐37 treatment inhibits the expression of inflammatory factors in Omicron‐infected M1 macrophages in vitro. (A) Experimental design and sample collection in vitro. (B and C) The relative mRNA expression of inflammatory factors in M1 macrophages, which were isolated from bone marrow, polarized into M1‐type macrophages, and subsequently infected with BA.5 or BF.7 following IL‐37 intervention. (D and E) The protein expression of inflammatory factors in M1 macrophages infected with BA.5 or BF.7 following IL‐37 intervention. *p < 0.05, **p < 0.01.

To further validate the anti‐inflammatory effect of IL‐37 administration on M1 macrophages during SARS‐CoV‐2 Omicron infection, we induced the differentiation of mouse bone marrow (BM)‐derived cells into M1 macrophages in vitro (Figure 5A). Given that there are numerous types of macrophage‐related cytokines, the results of the in vivo experiments revealed a significant decrease in CCL3, CCL4, etc., in both lung tissue and serum after IL‐37 treatment. For subsequent verification, we monitored the expression levels of CCL3 and CCL4. Our findings revealed a significant upregulation in the *Ccl3* and *Ccl4* mRNA expression in isolated and differentiated M1 macrophages 24 h after BA.5 infection. However, treatment with IL‐37 resulted in an approximately 20% reduction in the expression levels of these inflammatory factors (Figure 5B). Similarly, after BF.7 infection of M1 macrophages, the administration of IL‐37 led to an approximately 21% decrease in *Ccl4* mRNA expression and an approximately 15% reduction in *Ccl3* mRNA expression (Figure 5C). Furthermore, the cell supernatants were collected, and the production of inflammatory chemokines was determined. The results demonstrated a significant reduction in the production of CCL3 and CCL4 in the BA.5‐or BF.7‐infected M1 macrophages following the administration of IL‐37 (Figure 5D,E). These findings suggest that IL‐37 treatment exerts anti‐inflammatory effects by inhibiting the expression of inflammatory factors specifically in M1 macrophages.

### 2.6 IL‐37 Exerts an Anti‐Inflammatory Effect in Omicron Infection Through the Inhibition of the NF‐κB Signaling Pathway in M1 Macrophages

**FIGURE 6:**
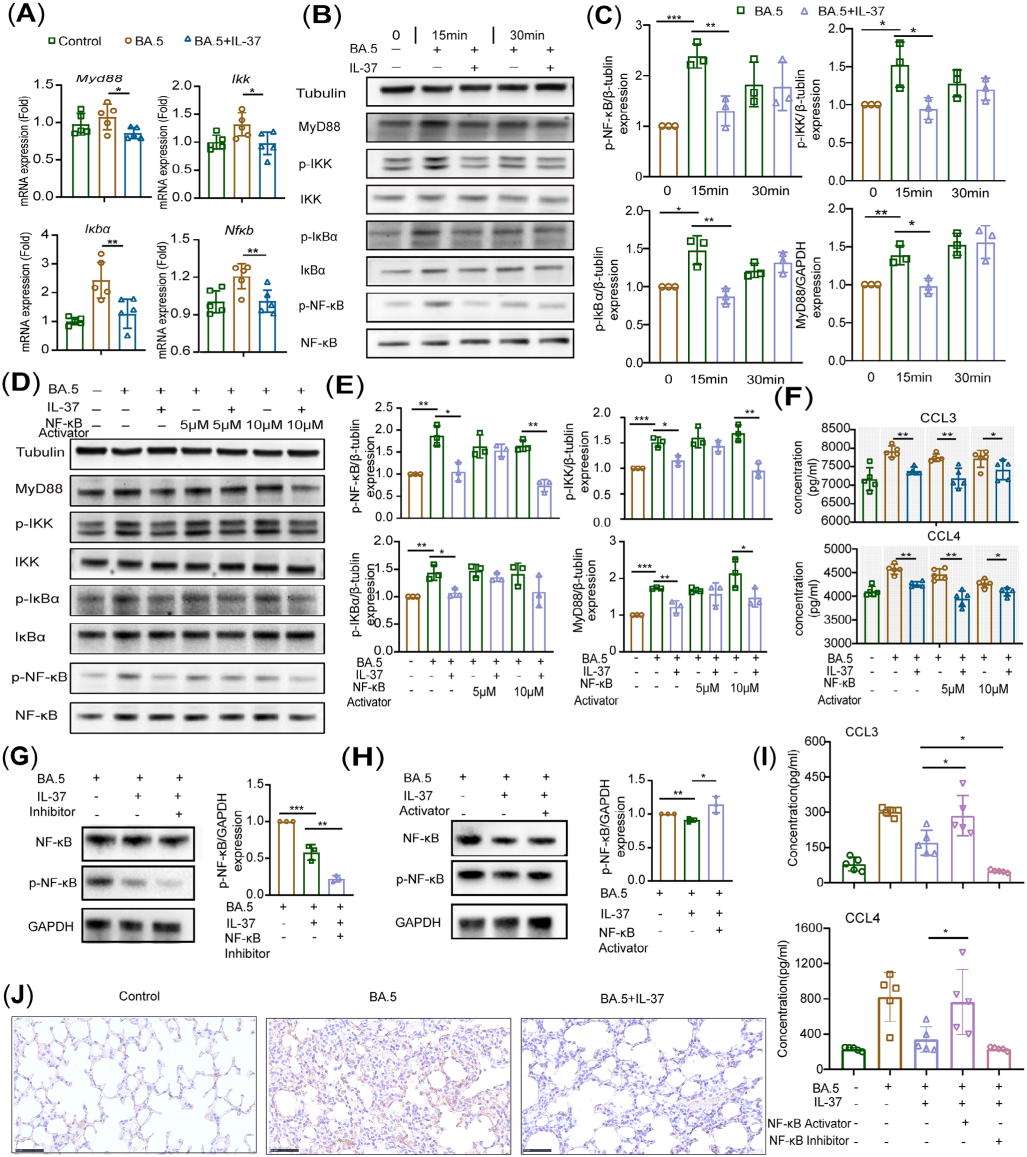
IL‐37 exerts an anti‐inflammatory effect on Omicron infection through the NF‐κB signaling pathway in M1 macrophages. The mRNA expression (A) and protein production (B) of the NF‐κB pathway‐related proteins in M1 macrophages infected with BA.5 were assessed after IL‐37 intervention. (C) Quantitative analysis of the results in (B). (D) Western blot analysis of the expression of NF‐κB pathway‐related proteins in BA.5‐infected and NF‐κB activator‐treated M1 macrophages using western blotting after IL‐37 treatment. (E) Quantitative analysis of the results shown in (D). (F) The protein expression of inflammatory factors in BA.5‐infected and NF‐κB activator‐treated M1 macrophages following IL‐37 intervention. (G) The level of p‐NF‐κB and NF‐κB proteins was shown and quantitatively analyzed in BA.5‐infected and NF‐κB inhibitor‐treated mouse bone marrow‐derived M1 macrophages was analyzed via western blotting after IL‐37 treatment. (H) The level of p‐NF‐κB and NF‐κB proteins was shown and quantitatively analyzed in BA.5‐infected and NF‐κB activator‐treated mouse bone marrow‐derived M1 macrophages was analyzed via Western blotting after IL‐37 treatment. (I) The production of inflammatory factors in BA.5‐infected and NF‐κB activator or inhibitor‐treated mouse bone marrow‐derived M1 macrophages following IL‐37 intervention. (J) NF‐κB expression in mouse lung tissue was detected via immunohistochemistry (magnification×400, black bar = 50 µm). *p < 0.05, **p < 0.01.

To further elucidate the underlying mechanism through which IL‐37 exerts its anti‐inflammatory effect on M1 macrophages during SARS‐CoV‐2 infection, we induced the differentiation of RAW264.7 cells into M1 macrophages. The findings revealed a substantial reduction in the mRNA expression of key pathway‐related proteins, including myeloid differentiation primary response protein 88 (MyD88), inhibitor of kappa B kinase (IKK), inhibitor of NF‐κB (IκBa), and NF‐κB, in M1 macrophages treated with IL‐37, further confirming its anti‐inflammatory effect on M1 macrophages through modulation of the NF‐κB pathway (Figure 6A). Furthermore, administration of IL‐37 reduced the expression of MyD88 and the phosphorylation of the IKK, IκBa, and NF‐κB proteins (Figure 6B,C).

To further verify the role of the IL‐37 signaling pathway in M1 macrophages, we infected M1 macrophages with Omicron BA.5 and subsequently stimulated the cells with varying concentrations of an NF‐κB activator. The results revealed a decrease in the expression of NF‐κB signaling pathway‐related proteins (MyD88, IKK, IκBa, and NF‐κB) in 10 µM NF‐κB activator‐stimulated and Omicron BA.5‐infected M1 macrophages after IL‐37 treatment (Figure 6D,E). Additionally, M1 macrophages infected with BA.5 presented elevated expression levels of chemokines such as CCL3 and CCL4; however, intervention with IL‐37 significantly reduced the expression of these inflammatory factors. Similarly, stimulation of M1 macrophages with an NF‐κB activator and subsequent infection with Omicron BA.5 resulted in increased production of the inflammatory factors CCL3 and CCL4; nevertheless, intervention with IL‐37 significantly attenuated the expression of these chemokines (Figure 6F). Furthermore, we differentiated mouse BM‐derived cells into M1 macrophages in vitro and introduced an NF‐κB activator or inhibitor to further elucidate the mechanism by which IL‐37 affects M1 macrophages during SARS‐CoV‐2 Omicron infection. As illustrated in Figure 6G, the level of phosphorylated NF‐κB was inhibited by an NF‐κB inhibitor, and the production of CCL3 and CCL4 was significantly decreased compared with that in the IL‐37‐treated and BA.5‐infected groups. Conversely, the addition of an NF‐κB activator led to a notable increase in phosphorylated NF‐κB (Figure 6H), along with a significant increase in the levels of CCL3 and CCL4 in the cellular supernatant (Figure 6I). In addition, we measured the NF‐κB expression in mouse lung tissue. Immunohistochemistry of lung tissue sections demonstrated a significant increase in NF‐κB expression in the infected group, which was effectively inhibited by the administration of IL‐37(Figure 6J). These results indicate that IL‐37 acts as an anti‐inflammatory factor capable of inhibiting NF‐κB pathway activation following the expression of macrophage‐related inflammatory factors.

## 3 Discussion

This study investigated the role of the anti‐inflammatory factor IL‐37 in Omicron variant infection‐induced pneumonia using a SARS‐CoV‐2 Omicron‐infected BALB/c mouse model. Mice infected with Omicron BA.5 and BF.7 experienced persistent weight loss, while lung histopathology revealed moderate interstitial pneumonitis characterized by perivascular and peribronchial infiltration of inflammatory cells. IL‐37 has a distinctive anti‐inflammatory effect against various pathogens and inflammatory diseases, including influenza virus, MERS‐CoV, coxsackievirus B3, gout, and rheumatic diseases. Three days post‐Omicron BA.5 or BF.7 infection, viral replication peaks, which coincides with substantial pathological damage to the lung tissue of the mice. Consequently, we chose 3 dpi to investigate the impact of IL‐37 on mitigating inflammation. We administered IL‐37 intravenously to SARS‐CoV‐2 Omicron‐infected mice. Following intervention with IL‐37, the body weights of the mice gradually recovered beginning at 2 dpi, and this recovery was accompanied by a decreased viral load, alleviated lung lesions, and reduced inflammatory cell infiltration. These findings suggest that IL‐37 effectively mitigates lung injury caused by Omicron infection. Additionally, the mRNA and protein expression levels of various inflammatory cytokines and chemokines, including CCL3, CCL4, and CXCL10, in both the serum and lungs of Omicron‐infected mice were significantly reduced following IL‐37 intervention.

Several studies have reported that, compared with those in the control group, the proportions of classical monocytes and monocyte‐derived macrophages in the BALF of COVID‐19 patients are greater, with the highest proportion being alveolar macrophages [24‐–26]. In our study, we found that the proportion of macrophages and the expression of some inflammatory factors in the lungs of mice infected with Omicron BA.5 or BF.7 increased, possibly because the SARS‐CoV‐2 infection of macrophages led to macrophages the release of many inflammatory factors. Inflammatory factors further stimulate macrophages to produce more inflammatory factors, forming a positive feedback loop, thereby driving persistent pulmonary infection. Notably, IL‐37 intervention markedly inhibited the local proportions of macrophages, particularly M1 macrophages, in lung tissue infected with Omicron, suggesting that IL‐37 may exert its anti‐inflammatory effects during Omicron infection through the modulation of macrophage secretion of related inflammatory cytokines and chemokines.

In addition, this study investigated the effects of the mode of administration on the anti‐inflammatory effects of IL‐37. Compared with those treated with intravenous peramivir, rats treated with inhaled peramivir have higher lung drug concentrations for longer duration and less systemic exposure [27]. In a piglet model of *Pseudomonas aeruginosa*‐induced pneumonia, compared with intravenous administration, nebulized administration of fucoxanthin results in greater drug deposition and lower bacterial counts in the lungs [28]. Similarly, compared with intraperitoneal injection, nebulized inhalation of the human monoclonal antibody 1212C2 results in reduced lung viral loads and decreased lung lesions in SARS‐CoV‐2‐infected hamsters [29]. Nebulization facilitates drug dispersion into fine droplets or particles that can enter the lungs through the respiratory tract, minimizing hepatic first‐pass effects and gastrointestinal degradation while increasing drug utilization efficiency [30]. This administration route is recommended for the treatment of various respiratory diseases, such as asthma and chronic obstructive pulmonary disease (COPD). Aerosolized administration directly targets the inflammatory site, specifically the respiratory mucosa, and exerts a rapid effect. In this study, nebulized IL‐37 was found to significantly decrease the production of CXCL9, IL‐17A, TNF‐α, and IL‐23 in both serum and lung tissues. Notably, while intravenous administration of IL‐37 did not result in a significant reduction of TNF‐α levels in the serum and lung tissues of mice, aerosolized IL‐37 treatment led to a marked decrease in TNF‐α levels in both compartments. We speculate that aerosolized IL‐37 administration may recruit a broader spectrum of immune cells to exert anti‐inflammatory effects via multiple inflammatory signaling pathways, thereby achieving superior anti‐inflammatory outcomes. However, further investigation into the additional immune cell responses following nebulized IL‐37 treatment in infected mice is warranted.

Studies have demonstrated the ability of SARS‐CoV‐2 to infect macrophages and induce their activation toward M1 macrophages [31]. M1 macrophages exhibit phagocytic activity against SARS‐CoV‐2, thereby facilitating viral replication and dissemination. In the lungs of SARS‐CoV‐2‐infected mice, predominantly M1 macrophages are observed; however, depleting these macrophages with clodronate attenuates lung pathology, suggesting that modulating the body's macrophage ratio and function could mitigate the inflammatory response [31]. In mice with myocardial infarction, IL‐37 intervention significantly decreases cardiac macrophage infiltration [32]. Similarly, IL‐37 intervention attenuates cigarette smoke‐induced inflammation in mouse lungs by reducing inflammatory cell infiltration and decreasing the proportion of alveolar lavage fluid macrophages [33]. Our findings demonstrated that IL‐37 treatment attenuated the proportions of M1 macrophages and the expression levels of proinflammatory cytokines and chemokines associated with macrophages, including IFN‐γ, TNF‐α, CCL2, CCL3, and CCL4, in the lung tissues of infected mice. These results suggest that IL‐37 may exert an anti‐inflammatory effect on coronavirus‐induced inflammation by modulating both the ratio and function of macrophages.

Macrophage‐induced inflammation is associated with the activation of various signaling pathways, including the mitogen‐activated protein kinase (MAPK), NF‐κB, and the Janus kinase/signal transducer and activator of transcription (JAK/STAT) pathways. Genes related to the NF‐κB pathway are upregulated in COVID‐19 patients, and severe cases exhibit increased NF‐κB activation [34]. The SARS‐CoV‐2 S protein triggers the activation of the macrophage NF‐κB signaling pathway and induces the expression of downstream proinflammatory cytokines and chemokines such as IL‐1β, CCL4, and TNF‐α [35]. Similarly, other respiratory viruses, such as respiratory syncytial virus (RSV) and SARS‐CoV, can also stimulate the NF‐κB signaling pathway. Significant activation of the NF‐κB pathway is observed in the lungs of BALB/c mice infected with RSV [36]. Additionally, infection of RAW264.7 cells with the SARS‐CoV S protein leads to a fivefold increase in NF‐κB activation [37]. Considering the association between the NF‐κB signaling pathway and inflammation, inhibiting its activation has proven effective in suppressing inflammation. Notably, anti‐inflammatory drugs such as dexamethasone and aspirin exert their effects by inhibiting the NF‐κB signaling pathway [38, 39]. Inhibitors targeting NF‐κB have been shown to enhance survival rates in mice infected with the recombinant adaptor strain of SARS‐CoV‐MA15 and reduce the production of inflammatory factors, including TNF‐α, CCL2, and CXCL2; moreover, they effectively attenuate lung injury in infected mice [40]. Furthermore, intravenous injection of NF‐κB inhibitors results in a significant decrease (90% for H1N1‐infected mice; 89% for H5N1‐infected mice; and 77% for H7N7‐infected mice) in lung viral titers [41]. The classic NF‐κB signaling pathway primarily responds to proinflammatory signals through cell surface receptors such as IL‐1R and TLR, which interact with adaptor proteins such as MyD88 and TRAF, subsequently activating IKK. This activation leads to the phosphorylation and degradation of IκB, resulting in the release of NF‐κB (a dimer of p50 and p65) for translocation into the nucleus. Consequently, the expression of inflammatory factors, including TNF‐α and CCL4, is upregulated [42, 43]. Our results revealed a decrease in the expression of NF‐κB pathway‐related proteins expression (MyD88, IKK, IκBa, and NF‐κB) in Omicron‐infected M1 macrophages following IL‐37 intervention. Furthermore, IL‐37 inhibited the production of these proteins in both NF‐κB activator‐treated and Omicron‐infected M1 macrophages. These findings suggest that IL‐37 treatment reduces inflammation during Omicron infection by inhibiting the classic NF‐κB signaling pathway, specifically in M1 macrophages. Additionally, lipopolysaccharide (LPS) stimulation activates the NF‐κB signaling pathway in mice; however, this activation is significantly reduced in abdominal macrophages from IL‐37 transgenic mice after LPS stimulation [22]. Moreover, IL‐37 can inhibit the translocation of NF‐κB to the nucleus and exert an anti‐inflammatory effect within the temporomandibular joint [44]. Another study demonstrated that through inhibition of the NF‐κB signaling pathway, IL‐37 treatment could protect against polarization toward M1 macrophages via inhibition of the NF‐κB signaling pathway [45]. These findings align with our findings and substantiate the ability of IL‐37 to modulate macrophage function and exert its anti‐inflammatory effects by targeting the intricate network involved in the regulation of NF‐κB signaling.

## 4 Conclusion

In summary, this study demonstrated that IL‐37 effectively mitigated symptoms in Omicron‐infected mice by suppressing proinflammatory cytokine and chemokine expression in the serum and lungs. Furthermore, IL‐37 alleviated lung inflammation by reducing the infiltration of inflammatory cells, particularly macrophages. These findings highlight the therapeutic potential of targeting the overactive immune response in SARS‐CoV‐2 infection‐induced pneumonia. Additionally, our study confirmed that M1 macrophages play a crucial role in mediating the anti‐inflammatory effects of IL‐37 during Omicron infection. We further revealed that the anti‐inflammatory effect of IL‐37 was exerted by inhibiting the MyD88/lκBa/NF‐κB pathway, specifically in M1 macrophages. In addition, this study has some limitations. The sampling period should be extended to 5–7 dpi to further clarify the role of M2 macrophages in this process, and the specific molecular mechanisms underlying the anti‐inflammatory effect of IL‐37 during Omicron infection should be further investigated. Moreover, it is essential to explore specific targets associated with the protective effect of IL‐37 against Omicron infection to provide fundamental insights for future clinical therapeutic strategies.

## 5 Materials and Methods

### 5.1 Virus and Mouse Experiments

The SARS‐CoV‐2 BA.5 strain (accession number: OP678016, SARS‐CoV‐2/human/CHN/GD‐5/2022) and the BF.7 strain (accession number: OQ372907, SARS‐CoV‐2/human/CHN/BJ‐12‐7/2023) were obtained from ILAS. Female specific‐pathogen‐free BALB/c mice aged 4–6 weeks were provided by Vital River (Beijing, China). Individual mice were intranasally inoculated with 50 µL of 10^5^ 50% tissue culture infectious dose (TCID_50_). The mice in the intravenous treatment group were subsequently intravenously injected with 100 µL of rh‐IL‐37 (12.5 µg/kg) at 2 h, 1 day, and 2 dpi. The mice in the aerosolized treatment group were administered aerosolized rh‐IL‐37 (10 µg/mL) for 5 min at 2 h, 1 dpi, and 2 dpi. The atomization rate was 0.28 mL/min, and the gas flow rate was greater than 10 L/min. The mice in the control group were intranasally administered 50 µL of PBS and treated with PBS at the same time points. The body weights and clinical symptoms of the mice were recorded daily, and serum and lung tissues were collected 3 days after infection.

### 5.2 qRT‒PCR

The experimental procedures are delineated in prior investigations [46, 47]. The sequences of the primers used for qRT‒PCR targeted the envelope protein (E) gene of SARS‒CoV‒2 and were as follows: forwards: 5′‐TCGTTTCGGAAGAGACAGGT‐3′, reverse: 5′‐GCGCAGTAAGGATGGCTAGT‐3′. The primer sequences for the individual genes encoding cytokines and chemokines are shown in Table S1.

### 5.3 Quantification of Cytokines and Chemokines

The lung tissue of the mice was processed by collection, fragmentation, and homogenization. After centrifugation, the supernatant was used for the detection of cytokines and chemokines using a mouse inflammation panel (13‐plex) kit and a mouse proinflammatory cytokine panel (BioLegend, 740446 and 740451).

### 5.4 Flow Cytometry Analysis

Lung tissues were dissected and digested to collect the single‐cell suspensions. Flow cytometry steps were performed according to previously described methods [18]. The cells were incubated with the following fluorescein‐conjugated antibodies: AF700‐anti‐CD45 (BioLegend, 157210), BV421‐anti‐F4/80 (BioLegend, 123137), PE/CY7‐anti‐CD11b (BioLegend, 101216), FITC‐anti‐CD80 (BioLegend, 104706), BB700‐anti‐CD86 (BD Biosciences, 742120), and BV785‐anti‐CD206 (BioLegend, 141729) and analyzed via flow cytometry (BD Biosciences), and the data were analyzed with FlowJo software.

### 5.5 BALF Analysis

BALF was obtained by washing the lungs of sacrificed mice twice with 1 mL of PBS and centrifuging them at 1500 rpm for 10 min at 4°C. The supernatant was collected and stored at −80°C. Total cellular infiltration in the BALF was quantified using a hemocytometer. Cytospin slides were prepared, fixed, and stained with Wright–Giemsa stain. The cellular composition was evaluated in a blinded manner by counting a minimum of 200 cells under a light microscope.

### 5.6 Pathological Examination

Paraffin sections (3, 4 µm in thickness) were deparaffinized and hydrated using xylene and an alcohol gradient and then stained with hematoxylin and eosin (H&E). Then, the sections were incubated with anti‐Mac‐2 antibody (Cedarlane, CL8942AP, 1:600) or anti‐NF‐κB antibody (CST, 6956S, 1:1000), then treated with FITC‐conjugated goat anti‐rat IgG (H+L) (Earthox, E031240, 1:200), or incubated with a goat anti‐rat IgG secondary antibody (HRP) (Beijing ZSGB Biotechnology, PV9004, 1:200), to assess the macrophage density using ImageJ software (NIH, Bethesda, MD, USA) and an Olympus microscope.

### 5.7 Polarization and Infection of Mouse BMDMs

Female BALB/c mice were euthanized and the tibias were collected to isolate BM cells. The BMs were cultured in RPMI 1640 culture medium supplemented with recombinant murine macrophage colony‐stimulating factor (M‐CSF, PeproTech, 315‐02) for 7 days. The cells were subsequently stimulated with 20 ng/mL M‐CSF, 20 ng/mL murine IFN‐γ (PeproTech, 315‐05), and 100 ng/mL LPS (Invitrogen, 00‐4976‐93) for 1 day. The cells were infected with BA.5 or BF.7 at a multiplicity of infection (MOI) of 0.01 in the presence or absence of IL‐37 (50 ng/mL). Both the cells and the supernatant were collected to assess the mRNA and protein expression levels of inflammatory factors. The treatment and experimental procedures for RAW264.7 cells mirrored those used for BMs.

### 5.8 Western Blotting

The experimental procedures followed those previously described [18]. Information on the antibodies used for western blotting is shown in Table S2. The protein bands were detected with a chemiluminescent imaging system (Amersham, Freiburg, Germany). The expression of a target protein was normalized to that of tubulin.

### 5.9 Statistical Analysis

The data were analyzed using GraphPad Prism version 8.0 through one‐way ANOVA test. *p* values < 0.05 were considered to indicate statistical significance (*p < 0.05, **p < 0.01).

## Author Contributions

Yiwei Yan, Feifei Qi, Mingya Liu, Fengdi Li, Ran Deng, and Xujian Liang conducted experiments, and acquired and processed the data. Feifei Qi, Yiwei Yan, Mingya Liu, Qi Lv, Yanfeng Xu, Ming Liu, Shuyue Li, and Guocui Mou contributed to the data analysis and interpretation. Feifei Qi and Yiwei Yan wrote the manuscript. Linlin Bao and Feifei Qi contributed to the conception and design. All authors ultimately approved the manuscript. All authors approved the manuscript for submission.

## Ethics Statement

The animal experiments were performed in an animal biosafety level 3 (ABSL‐3) laboratory at the Institute of Laboratory Animal Science (ILAS). All procedures in this study involving mice were evaluated and approved by the Institutional Animal Care and Use Committee (IACUC) of the ILAS (BLL22012).

## Conflicts of Interest

The authors declare no conflicts of interest.

## Supporting information



Supporting Information

## Data Availability

All data and materials related to this paper are available upon request.
