# Supplementary material for: IL‐37 Mitigates the Inflammatory Response in Macrophages Induced by SARS‐CoV‐2 Omicron Infection Through the NF‐κB Signaling Pathway
Source: MedComm (2020). 2025 May 31;6(6):e70229. doi: 10.1002/mco2.70229 (PMC12126598; doi:10.1002/mco2.70229)
Supplement: Supplementary file 1 — Supporting Information [file MCO2-6-e70229-s001.docx]

**Title page：**

**Type of study: Original Research Article**

**IL-37 mitigates the inflammatory response in macrophages induced by SARS-CoV-2 Omicron infection through the NF-κB signalling pathway**

**Short Running title: IL-37 mitigates inflammation in Omicron infection**

**Feifei Qi^1,2,#^, Yiwei Yan^1,#^, Mingya Liu^1,#^, Qi Lv^1,2^, Yanfeng Xu^1,2^, Ming Liu^1^, Fengdi Li^1,2^, Ran Deng^1,2^, Xujian Liang^1,2^, Shuyue Li^1^, Guocui Mou^1^, Linlin Bao^1,2,3*^**

^1^ Beijing Key Laboratory for Animal Models of Emerging and Reemerging Infectious Diseases, NHC Key Laboratory of Comparative Medicine, Institute of Laboratory Animal Science, CAMS &PUMC, Beijing, 100021, China

^2^ National Center of Technology Innovation for Animal Model, Beijing, 100021,China

^3^ State Key Laboratory of Respiratory Health and Multimorbidity, Beijing, 100021, China

^#^ These authors contributed equally to this work.

^*^ **Corresponding author**

Linlin Bao, Institute of Laboratory Animal Sciences, Chinese Academy of Medical Sciences (CAMS) & Comparative Medicine Center, Peking Union Medical Collage (PUMC), Beijing, China.

Email: [bllmsl@aliyun.com](mailto:bllmsl@aliyun.com)

**Table S1 Primer sequences**

| **Gene** | **Forwards（5’-3’）** | **Reverse（5’-3’）** |
| --- | --- | --- |
| *β-actin* | CAACGAGCGGTTCCGATG | GCCACAGGATTCCATACCCA |
| *Il-6* | TCTATACCACTTCACAAGTCGGA | GAATTGCCATTGCACAACTCTTT |
| *Ccl3* | TTCTCTGTACCATGACACTCTGC | CGTGGAATCTTCCGGCTGTAG |
| *Ccl4* | TTCCTGCTGTTTCTCTTACACCT | CTGTCTGCCTCTTTTGGTCAG |
| *Cxcl10* | CCAAGTGCTGCCGTCATTTTC | GGCTCGCAGGGATGATTTCAA |
| *Ifng* | TATCTGGAGGAACTGGCAAA | GGTGTGATTCAATGACGCTT |
| *Ccl2* | AGTAGGCTGGAGAGCTACAA | GTATGTCTGGACCCATTCCTTC |
| *Cxcl9* | TCCTTTTGGGCATCATCTTCC | TTTGTAGTGGATCGTGCCTCG |
| *Il-1α* | TCTCAGATTCACAACTGTTCGTG | AGAAAATGAGGTCGGTCTCACTA |
| *IL-1β* | CAACCAACAAGTGATATTCTCCATG | GATCCACACTCTCCAGCTGCA |
| *Tnf-α* | CCTCTCTCTAATCAGCCCTCTG | GAGGACCTGGGAGTAGATGAG |

**Table S2 Western blotting antibodies**

| **Name** | **Company** | **Cat** |
| --- | --- | --- |
| β-Tubulin (D2N5G) Rabbit mAb | CST | 15115 |
| β-Tubulin (D3U1W) Rabbit mAb | CST | 86298 |
| MyD88 (D80F5) Rabbit mAb | CST | 4283 |
| Phospho-IKKα/β (Ser176/180) (16A6) Rabbit mAb | CST | 2697 |
| IKKβ (D30C6) Rabbit mAb | CST | 8943 |
| Phospho-IκBα (Ser32)(14D4) Rabbit mAb | CST | 2859 |
| IκBα(44D4) Rabbit mAb | CST | 4812 |
| Phospho-NF-κB p65 (L8F6) Mouse mAb | CST | 3033 |
| NF-κB p65 (Ser536) (93H1) Rabbit mAb | CST | 6956 |
